# Elements of the Comparative Anatomy of the Vertebrate Animals

**Published:** 1846-04

**Authors:** 


### Elements of the Comparative Anatomy of the Vertebrate Animals, designed especially for the Use of Students. By *Rudolph Wagner*, M.D., Professor of Comparative Anatomy and Physiology in the University of Göttingen, &c. &c. Edited from the German, by *Alfred Tulk*, Member of the Royal College of Surgeons of England. Octavo, pp. 264. London: Longman & Co. 1845.

The peculiar advantage of Comparative Anatomy is, that by the extended range of its inquiries, and by the number of its facts, it reveals to the student the fundamental laws of organization, and thus enables him to detect principles of formation which are masked beneath the complexity of human anatomy. Combined with embryology, it also establishes in the fullest extent the universality of typical forms, and proves by evidence that cannot be mistaken the unity which pervades the animal creation. But to produce results of this magnitude, comparative anatomy must be cultivated in a connected manner and in an enlarged spirit; that is to say, the same system of organs must be examined in different animals; points of resemblance and diversity must be scrupulously noted; unknown forms must be compared with those which are known, so that their true character and relations may be seized and determined; and more than all it is essential, by a rigid system of mental training, to acquire that acumen which, in the midst of seeming resemblances and apparent differences, enables the observer to discern the truth and to enunciate the great principles of animal formation. The Hunterian Museum is not only valuable on account of the matchless specimens it contains, but more especially because, by *its plan*, it has taught zoologists the true end and scope of comparative anatomy. This plan consists in exhibiting, by an extensive series of preparations, the forms and structure of each particular class of organs, those of digestion for instance, tracing them from their most simple to their most complex development, and thus following them in a connected manner from the lowest to the highest animal; a mode of procedure which has since been adopted and extended in the admirable Leçons of Hunter's illustrious successor, Cuvier.

The scope of the work before us is however of a more limited character; it aspires only to present a kind of catalogue raisonnée of the individual organs as they exist in the vertebrata. A manual of this kind, accurate as to its facts, and judicious in its arrangement, is nevertheless a most useful production, serving to introduce the student to what is in itself unquestionably one of the most interesting branches of human research. The deservedly high reputation of Professor Wagner is a guarantee that the indispensable qualities we have noticed will not be wanting; and we are for these reasons glad to find that Mr. Tulk has presented to the English reader a translation of his "Lehrbuch der Zootomie," which is in fact a new edition of the author's earlier work, entitled "Lehrbuch der Vergleichenden Anatomie." The reasons which have prompted Mr. Tulk in his selection are thus explained:—"The want of some good elementary work in our own language, at least of one that, within a small compass and reasonable price, should express the amount of our knowledge upon the Anatomy of the several classes of Vertebrate Animals, was the chief inducement to my undertaking the translation of the present work, in the selection of which I was encouraged by the very favourable light in which Dr. Rudolph Wagner had been already made known to the Medical Profession through his 'Elements of Physiology,' so ably edited by Dr. Willis. The thorough practical knowledge which he possesses of his subject, combined with the clearness and brevity of his style, render indeed his works the very best introductions that are extant to the sciences of Physiology and Comparative Anatomy."—*Advertisement.*

In noticing a work of this elementary character, we cannot of course give any general views on animal organization; we must therefore content ourselves with laying before our readers a few detached extracts descriptive of some of the more interesting among the various organs, which will serve at the same time to convey an idea of the scope of Dr. Wagner's manual.

The details respecting the vascular system are, for a work of this nature, ample, and the student will find, as in other parts of the treatise, many new facts introduced. The careful investigation of the heart and great vessels as they exist in reptiles, affords many important elucidations respecting the malformations of the same parts in the human body. Thus we find in the chelonia that the ventricle apparently single on the exterior is internally divided into two cavities by a septum, which is however more or less perforate; whilst, in crocodiles, the two ventricles are completely separated by a strong septum from each other, though a partial admixture of the venous and arterial blood still takes place in consequence of a communication between the two great arterial trunks. Conditions like these are occasionally seen in malformations; and it is interesting to remark, which we have ourselves often done, that when the two ventricles of the human heart communicate, the perforation is at the base or upper part of the septum cordis, or precisely at that part which is the last to be closed up both in reptiles and in the development of the heart in birds and mammals.

The veins of the abdomen in the three lower classes of the vertebrata offer very important evidence as to the true character of the renal secretion. In all these animals, that is to say, in birds, reptiles, and fishes, the portal system is double, one part belonging to the liver, and the other to the kidney; the former, or hepatic, consisting more especially (and this is an interesting distinction) of the splenic and mesenteric veins returning the blood from the digestive organs; whilst the latter, or renal, more varied in its formation, receives its blood, in a general way, from the pelvis, from the posterior extremities when these exist, and from the walls of the abdomen. There is thus in all these classes a peculiar set of vessels going to the kidney, in addition to the emulgent artery and vein; the signification of which arrangement was first expounded by Mr. Bowman, in his admirable work on this organ. Without dwelling upon details which would be here misplaced, we will only remark that the large *afferent* or reno-portal vein, usually situated, as in the frog, towards the outer and posterior part of the kidney, penetrating the organ, divides into branches, which, uniting with the *efferent* vessels of the Malpighian bodies, form an intricate capillary plexus displayed over the walls of the urinary tubules, and doubtless engaged in the act of secretion: the *efferent* vein of the kidney is formed by the terminal branches of the above plexus, and thus returns to the system of the inferior cava, the residual blood carried to the organ, both by the renal artery and by the afferent vein. It is in this manner proved that a part at least of the urinary secretion is derived, like the bile, from venous blood; a fact of the deepest interest as regards the metamorphoses of the body, and which, it is not too much to assert, could never have satisfactorily been determined without the aid of comparative anatomy.

The vascular system offers many other remarkable modifications which throw much light on the powers concerned with the circulation of the blood and lymph. One of these consists of what are called lymphatic hearts, and of which the following is a good description.

"The pulsating *lymphatic hearts*, which have been discovered chiefly in the ischiadic region of the Frogs, Salamanders, Serpents, Tortoises and Crocodiles, constitute one of the most remarkable peculiarities of the present system in Reptiles. They are provided with muscular walls, the fibres composing which exhibit under the microscope the peculiar transverse striæ characteristic of the voluntary muscular tissue. The posterior pair of these hearts may be seen externally pusating very distinctly immediately beneath the integument covering the ischiadic region in the Frog; they discharge their contents into a branch of the ischiadic vein; situated more deeply above the third cervical vertebra, lie the anterior lymphatic hearts in the same animal, and they appear to impel their contained fluid into a branch that opens into the jugular vein. These organs appear to be largest in the Chelonia, where, placed invariably behind the superior extremity of the iliac bones upon the origin of the semitendinosus muscle, they measure an inch in diameter, and receive lymphatic vessels of the thickness of a quill; they pour their lymph into a vein that forms a twig of the reno-portal vein." (*L. c.*, p. 168.)

The lymphatic system of fishes, known since the time of the Hunters and Monros, is said to have a more extensive distribution than was hitherto suspected. Professor Wagner observes,

" Peculiar *Caudal* and *Cranial sinuses* and a special *system of Lateral vessels* were recently discovered in Fishes, and seem to belong to the lymphatic system. Beneath what has been formerly described as the lateral or mucous canal of the integument, we find another sinus-like canal that is filled with a clear and limpid lymph, and communicates with a number of adjacent branches, all pursuing a subcutaneous course, and forming a ring of vessels around each scale, so that the body of Fishes is completely intersected by this net-work. This system of vessels communicates with a peculiar caudal sinus, that is placed in many Fishes, as the Eel, in the same situation as the caudal heart, which will presently be described. This sinus is double, the divisions lying close upon either side of the flat rays supporting the caudal fin; but both of them communicate by means of a transverse canal that passes through an opening in one of the fin-rays. The sinus varies in size, and passes into the caudal vein, being there provided with a valve. It is invested by a strong fibrous tunic, and contains a clear lymph: whether it is endowed with powers of expansion and contraction has not yet been determined. A similar sinus has been observed upon either side the cranial cavity external to the jugular veins; it is pear-shaped, smaller than the caudal sinus, and appears to be contractile. The occurrence and position of these reservoirs remind us very much of the lymphatic hearts of the Amphibia." P. 242.

The caudal heart of the eel above alluded to propels the blood into the caudal vein: it is double, is placed on each side of the last caudal vertebra, and can be seen in action by holding up the tail of the living animal to the light: it is said to occur also in muræenophis. Besides these venous cardiac appendages, there are even accessory hearts connected with the arteries in some fishes: " thus in chimæra an elongated fusiform *accessory heart* is always developed upon the two axillary arteries destined to supply the pectoral fins. Similar axillary hearts also occur in Torpedo, but not in Raia."

The most numerous of these cardiac appendages are, however, met with in that anomalous animal the Amphioxus Lanceolatus or Lancelet, which, although a vertebrate creature, has quite colourless blood without corpuscles, and in which no distinct evidence of a brain has been detected. Besides the ordinary arterial heart (which wants the pericardium), there are bulbilli representing the branchial arteries; contractile aortic arches; a long tubular portal heart on the ventral side of the intestine; and, lastly, a heart of the vena cava, which lies on the opposite or dorsal side of the intestine. These venous hearts contract alternately, and the whole vascular system, according to the author, recals that in the annelida, where numerous pulsating heart-like vessels also occur.

The section treating of the vocal apparatus in birds we have perused with much pleasure. We need scarcely remind our readers that, in this division of the animal kingdom, with a few limited exceptions where the voice is entirely absent, there are a superior and an inferior larynx, the latter of which is unquestionably the true vocal organ, and consequently presents the greatest variety of structure in different birds. The superior larynx, situated as usual behind the tongue, is a sufficiently complex organ, consisting of from four to ten osseous or cartilaginous pieces, corresponding, according to Wagner, to the cartilages of the human larynx, and acted upon by three pairs of muscles which are constant. After pointing out the great length of the trachea, which is composed of from 20 to 70 rings (in passerine birds), to one, two, and even three hundred rings (among the natatores, grallæ and struthionidæ), and noticing the remarkable spiral convolutions often met with in this tube, either placed within the keel of the sternum or in its vicinity, the author thus describes the true vocal apparatus.

" The existence of an *Inferior* or *Bronchial Larynx*, in which the voice is produced, constitutes a special peculiarity of the class of Birds. It is situated in the upper part of the thoracic cavity at the extremity of the trachea, where that divides into the two bronchi. The length of the inferior larynx is mostly greatest from before backwards, and its interior forms a quadrangular cavity, which is generally divided inferiorly (at its outlet) by a *cross-bone* passing from before backwards, into two lateral halves, that receive the two openings of the bronchi, which are to be viewed as forming a double rima glottidis. The cross bone consists of the last tracheal ring increased in size, or else of several rings which have become firm and rigid, approximated and even blended together, so as to form a firm bony drum of variable size. The commencement of the bronchial tubes is also to be regarded as forming part of the inferior laryngeal apparatus, since their first rings and the membranes which unite them must exert the greatest influence upon the production of the voice."

" In the majority of those Birds which are capable of uttering sounds membranes are found situated both exteriorly and internally to the inferior larynx. There arises from the cross-bone a thin membranous slightly elastic and easily lacerable membrane, which completes the bronchi upon their internal aspect. The extent of this membrane is greater or less in proportion to that of the segment formed by the imperfect bronchial rings; the first two or three of these are usually very slightly curved, and in the form only of a small semicircle; the membrane completing the rings is therefore largest in this situation, and fully merits its appellation of *membrana tympaniformis interna*. There is generally found a *membrana tympaniformis externa* presenting the form of a fenestroid oval membrane placed between the cross-bone or the lowermost tracheal ring and the most superior of the bronchial semicircular cartilages." P. 112.

Several modifications of the organ are described, but which our limits will not allow us to notice; we therefore pass on to the larynx of singing-birds, possessing what is called a true *muscular vocal apparatus*, and which can also be well studied in the raven or rook.

" The trachea here consists of a short bony tympanum or drum, constituting its inferior extremity, and usually formed by the early coalescence of three rings. The tympanum is divided as usual inferiorly by a transverse or cross bone. From the superior border of this, a membranous semilunar fold (*membrana semilunaris Savart*) rises to about a line in height, as in the Rook; its dimensions, however, are very various, and in birds of accomplished song, or such as can learn to speak, it is more largely developed. In other Passeres, as in the Sparrow, Haw-grosbeak, &c. these membranes are of course wanting. From these observations it may be inferred that this membrane is of essential importance to the production of song or the power of uttering articulate sounds, since when it is absent or slightly developed, the voice is feeble or has but little variety of tone. The three first bronchial demi-rings exhibit also remarkable peculiarities. The first or uppermost ring is thicker in the middle than at the extremities; at the posterior end inferiorly it becomes broader, and curves in such a manner backwards and inwards, as to form the posterior and internal wall of the bronchial portion of the inferior larynx; below it passes into an acute angle, which forms the point of support of the internal lip of the glottis. The second bronchial demi-ring is more moveable than the first, especially in the direction outwards and upwards. The third demi-ring is nearly straight, and between it and the second demi-ring there is a triangular membranous fenestra, and between the first and second bronchial demi-ring an oval *membrana tympaniformis externa.* Upon its inner surface there is found a remarkable fold of the mucous membrane, consisting of tolerably thick and elastic tissue; it forms a true vocal chord, *ligamentum vocale externum,* which always forms the external lip or wall of the glottidean fissure. This membrane or fold is thrown into vibrations during the passage of the air from the lungs. Besides this the *membrana tympaniformis interna* is found as usual completing the inner side of the bronchi, and is in continuous connection with the *membrana semilunaris* of the cross-bone. A small pliant cartilage (*cartilago arytænoidea Savart*) is situated in the upper part of this membrane, is in connection with the second arch of bone, and exhibits manifold diversities." P. 115.

Acting upon this complex mechanism, there are no less than five pairs of intrinsic muscles, in addition to two external pairs connected with the trachea. "The voice of birds appears like that of the human subject to be produced by the combined vibrations of the laryngeal membranes and the tongue. The flute-like tones of the Singing-birds are doubtless accomplished by a vibration of the whole column of air while passing through the trachea."

Another interesting appendix of the respiratory organs, for such we regard it to be, is the *swimming-bladder* of the fish. The author remarks—

"Frequently as it has been compared with the lungs of the higher Vertebrata, and certainly, from its mode of development, position, and internal structure, reminding us exceedingly of these organs in the Amphibia, still the disposition of its vessels forbids our regarding the *Swimming-bladder* as an instrument of respiration, and thus we are still in doubt as to its precise functions; it occurs only in the Bony Fishes, but not in all the genera, and among the Cartilaginous in the Sturgeon alone, which forms the transition-link to the Osseous Fishes. The Swimming-bladder must however exist in connexion with definite modes of life in several Fishes, since it is frequently absent in different species of a genus, or in nearly allied genera, *e. g.* Scomber scombrus, Polynemus paradiscus, and the genera Pleuro-nectes and Lophius.

"In ordinary cases the swimming-bladder is situated beneath the spinal column, to which it is firmly attached by cellular tissue, and, covered by the kidneys, overlaps the intestinal canal. It consists of two coats; of an external, which is very tough, fibrous, and glistening, and an internal or soft vascular mucous membrane. It is invested upon its lower surface, or that facing the viscera, by peritoneum." P. 250.

That this remarkable organ is to be regarded as a rudimentary lung, cannot, we think, be reasonably doubted. The greatest obstacle to the reception of such an opinion is, that fishes are destined to breathe by gills and not by lungs; but there are not wanting examples to prove that animals respiring essentially by branchiæ, such as the proteus, siren, and axolotl, may still possess lungs. The above theory rests, however, on the more certain basis of embryology, which proves that all vertebrate animals, whatever may be the ultimate mode of their respiration, present in their development the types both of branchial and pulmonary organs; so that in fact, instead of the existence of rudimentary lungs in fishes being an anomaly, the real exception would have been, if such organs had at no period of their formation existed.

Although the homology of the swimming-bladder may be thus determined, it is by no means easy to assign its use. That it does not in general act as a lung is certain; for, among other reasons which might be adduced, it will suffice to state that this bladder is often a closed sack, though at other times it has an external communication by what has been called its ductus pneumaticus. Nor is it sufficient to affirm that it is employed exclusively as a pneumatic apparatus, enabling the fish to rise or sink according as the air within is expanded or compressed by the action of the muscles attached to it, of which one or more pairs have been described. The great vascularity of the inner membrane, which can be beautifully seen by the microscope in the eel, clearly indicates some additional and important function; a position which is further supported by the fact that in many instances a peculiar *vascular gland* is interposed between the coats of the swimming bladder, presenting a very curious arrangement of blood-vessels.

Our limits will only permit us to extract the following account of the complicated muscular structure connected with the skin of birds, the representative of the panniculus carnosus of quadrupeds. "The tegumentary muscles which contract the skin are very extensively developed in the present class, as also the muscles which bristle up the feathers upon the neck and head particularly of those birds, where they arise in tufts. In the Gallinæ a peculiar tegumentary muscle supports the crop as it hangs down in the neck. The strongest tegumentary muscles occur in the Apteryx, in which several distinct layers and fasciculi may be distinguished; a provision very necessary to this bird, which scratches deep in the earth, and must therefore shake its feathers with considerable force to dislodge the dirt from them. In addition to these muscles in birds, there are strips appropriated to the feathers, which becoming detached from the tegumentary muscles, form a sheath around the quill part of each feather where it projects into the skin. They are wanting generally to the down-feathers, and are in the others easily overlooked on account of their small size in most birds. In the larger birds, however, as some of the Palmipedes, *e. g.* the Pelican, Goose and Duck, they are very much developed, each feather receiving four, more rarely five, small muscular fasciculi, which can move the feather in all directions, so that in Sula and Anas, where about 3000 quill-feathers are reckoned upon the body, the number of these muscles amounts to 12,000." (*L. c.* p. 83.)

We must here conclude our notice of Professor Wagner's interesting and valuable work. But we cannot do so without offering our thanks to Mr. Tulk for the judicious selection he has made, and for the acceptable service he has thus rendered to the student of comparative anatomy. We would further express our hope that this gentleman will receive sufficient encouragement to complete this outline of animal organization, by translating those parts of Professor Wagner's work, which treat of the invertebrate animals, and which are now being published in Germany, under the superintendance of the author by two of his pupils, Dr. Heinrich Frey and Dr. Rudolph Leuckart. The part comprising the Insecta has lately reached this country. There is also one other addition, which we trust the translator may be enabled to make, and that is an atlas of plates, without which it is almost impossible to convey accurate conceptions of structure to those who, like so many of our professional brethren, have not access to museums of comparative anatomy.

